# Author Correction: Electrochemical and hot corrosion behaviour of annealed AlCoCrFeNi HEA coating over steel

**DOI:** 10.1038/s41598-024-75819-x

**Published:** 2024-10-25

**Authors:** N. Radhika, Niveditha Noble, Adeolu Adesoji Adediran

**Affiliations:** 1 Department of Mechanical Engineering, Amrita School of Engineering, Amrita Vishwa Vidyapeetham, Coimbatore, India; 2 Department of Mechanical Engineering, Landmark University, P.M.B. 1001, Omu-Aran, Kwara State Nigeria; 3 Department of Mechanical Engineering Science, University of Johannesburg, Auckland Park Kingsway, Johannesburg, South Africa

Correction to: *Scientific Reports* 10.1038/s41598-024-55962-1, published online 07 March 2024

The original version of this Article contained an error in Figure 17(e) and Figure 18(b), where the wrong figures were uploaded.

The original Figure 17 and Figure 18, and accompanying legends appear below.

**Fig. 17:**
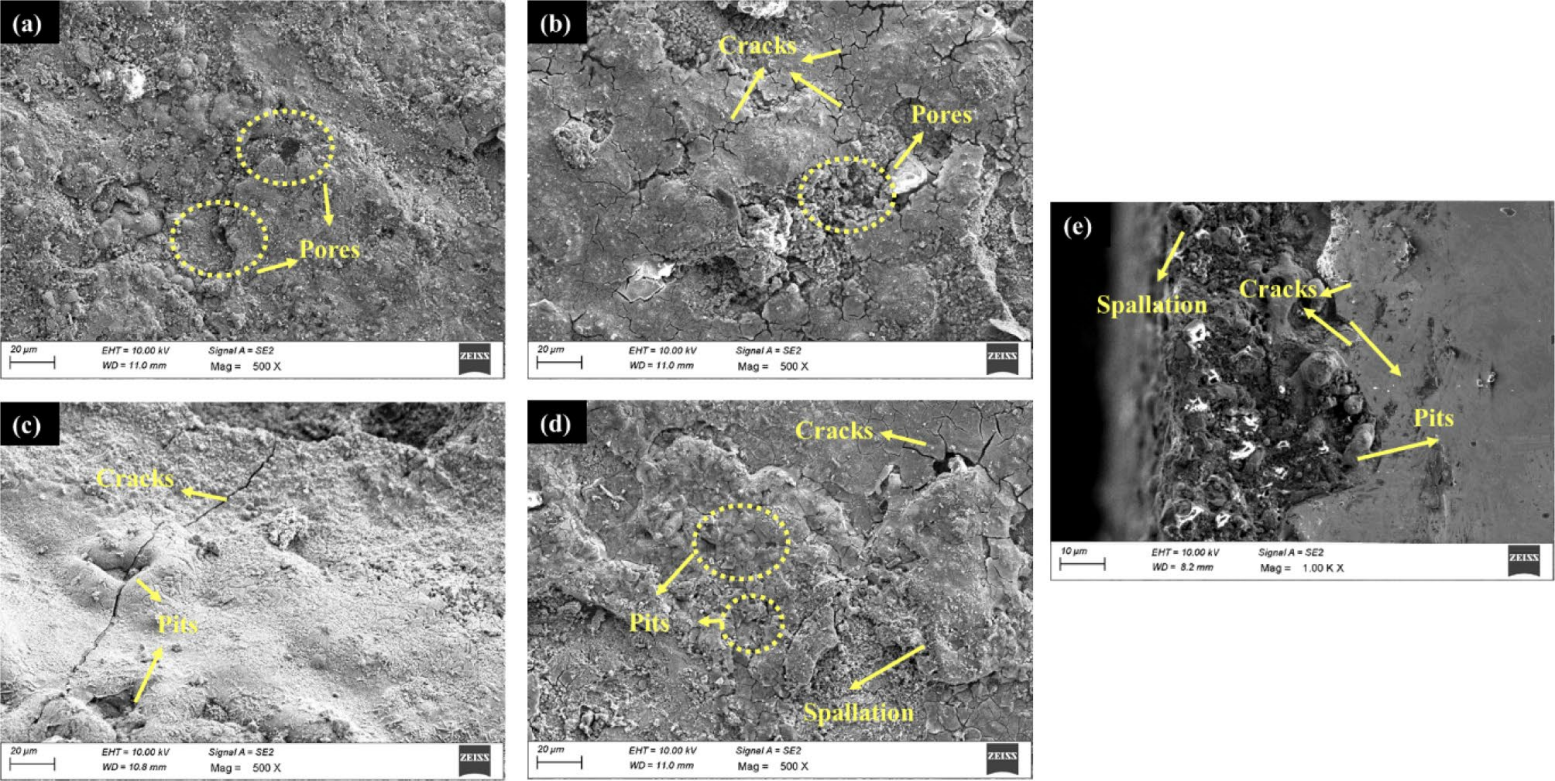
SEM images of the hot corroded surface under mixture C: (**a**) uncoated sample (**b**) uncoated sample post annealing (**c**) coated sample (**d**) coated sample post annealing (**e**) cross section of coated sample post annealing.

**Fig. 18:**
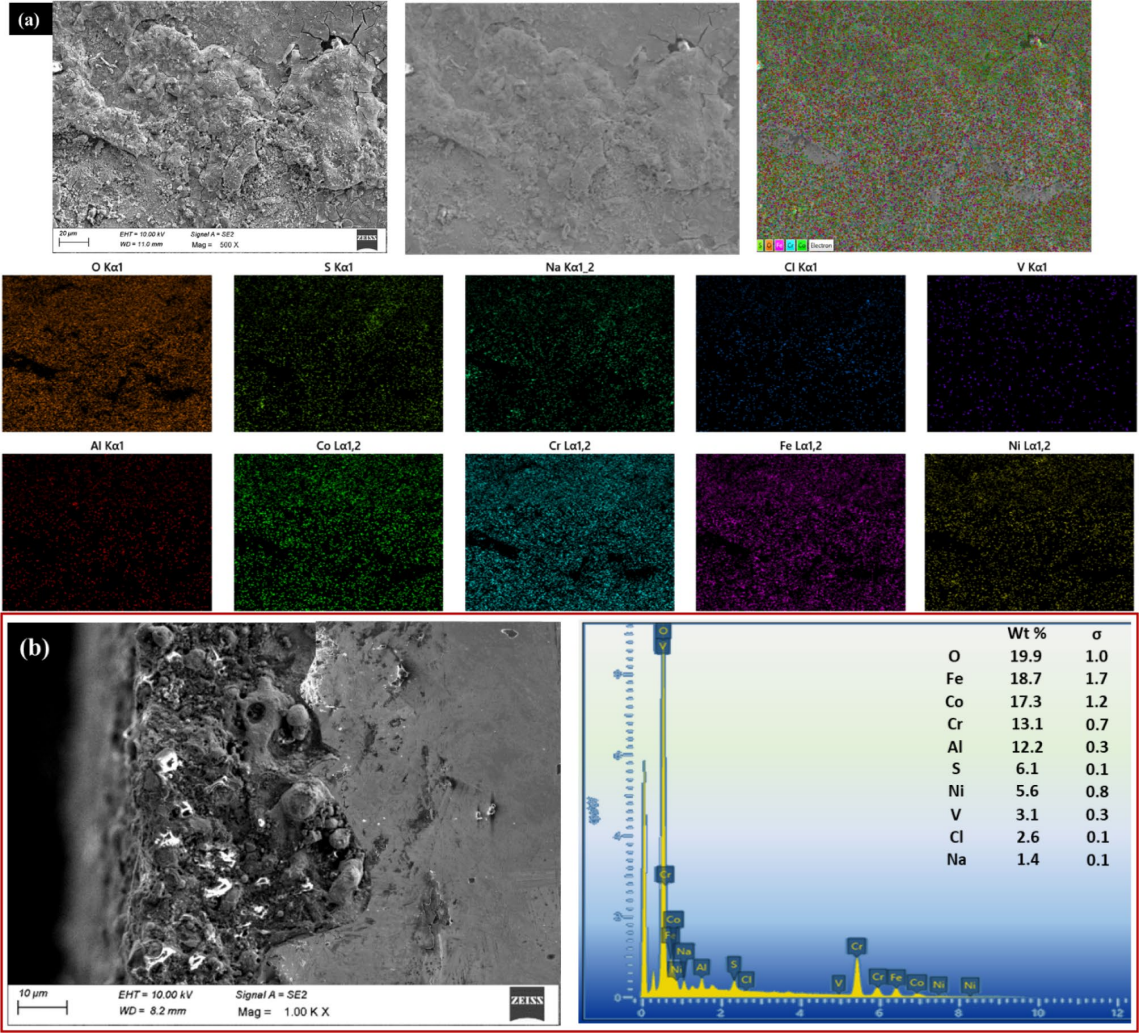
(**a**) Elemental mapping (**b**) EDS mapping of the cross section of the coated and annealed surface corroded under salt mixture C.

The original Article has been corrected.

